# Lactational Changes of Phospholipids Content and Composition in Chinese Breast Milk

**DOI:** 10.3390/nu14081539

**Published:** 2022-04-07

**Authors:** Meng-Tao Yang, Qiu-Ye Lan, Xue Liang, Ying-Yi Mao, Xiao-Kun Cai, Fang Tian, Zhao-Yan Liu, Xiang Li, Yan-Rong Zhao, Hui-Lian Zhu

**Affiliations:** 1 School of Public Health, Sun Yat-sen University, Guangzhou 510080, China; yangmd7@mail2.sysu.edu.cn (M.-T.Y.); lanqy6@mail.sysu.edu.cn (Q.-Y.L.); liuzhy235@mail.sysu.edu.cn (Z.-Y.L.); 2 Guangdong Provincial Key Laboratory of Food, Nutrition and Health, Guangzhou 510080, China; 3 School of Life Sciences, Beijing University of Chinese Medicine, Beijing 102401, China; liangx75@mail2.sysu.edu.cn; 4 Abbott Nutrition Research & Development Center, Abbott Ltd., Shanghai 200233, China; yingyi.mao@abbott.com (Y.-Y.M.); xiaokun.cai@abbott.com (X.-K.C.); fang.tian@abbott.com (F.T.); xiang.li@abbott.com (X.L.)

**Keywords:** Chinese breast milk, phospholipids, lactational stages, MUAI, HPLC-ELSD

## Abstract

Phospholipids are pivotal polar lipids in human milk and essential for infants’ growth and development, especially in the brain and cognitive development. Its content and composition are affected by multiple factors and there exist discrepancies in different studies. In this study, we determined five major phospholipids classes (phosphatidylethanolamine, phosphatidylinositol, phosphatidylserine, phosphatidylcholine, and sphingomyelin) in 2270 human milk samples collected from 0 to 400 days postpartum in six regions of China. The high-performance liquid chromatography coupled with an evaporative light scattering detector (HPLC-ELSD) was performed to quantify the phospholipids. Total phospholipid median (IQR) content was in a range between 170.38 ± 96.52 mg/L to 195.69 ± 81.80 mg/L during lactation and was higher concentrated in colostrum milk and later stage of lactation (after 200 days postpartum) compared with that in the samples collected between 10 to 45 days postpartum. Variations in five major sub-class phospholipids content were also observed across lactation stages (phosphatidylethanolamine: 52.61 ± 29.05 to 59.95 ± 41.74 mg/L; phosphatidylinositol: 17.65 ± 10.68 to 20.38 ± 8.55 mg/L; phosphatidylserine: 15.98 ± 9.02 to 22.77 ± 11.17 mg/L; phosphatidylcholine: 34.13 ± 25.33 to 48.64 ± 19.73 mg/L; sphingomyelin: 41.35 ± 20.31 to 54.79 ± 35.26 mg/L). Phosphatidylethanolamine (29.18–32.52%), phosphatidylcholine (19.90–25.04%) and sphingomyelin (22.39–29.17%) were the dominant sub-class phospholipids in Chinese breast milk during the whole lactation period. These results updated phospholipids data in Chinese human milk and could provide evidence for better development of secure and effective human milk surrogates for infants without access to breast milk.

## 1. Introduction

Human milk is the gold standard source of nutrition at the onset of life, and exclusive breastfeeding is advocated for newborns in the first 6 months [1]. Comprising macronutrients, adequate micronutrients and bioactive molecules, human milk is known to benefit infants to meet their nutritional needs [2], promote optimal development [3], and also reduce the risk of infections and later allergies [4]. Milk lipids are an important component in human milk, providing infants with about 50% of the calories needed for postnatal growth and development [5], and the usual form of which is milk fat globule (MFG). The lipid droplets are surrounded by a coating consisting of triple phospholipids (PLs) and a cholesterol layer with incorporated proteins and glycoproteins deriving from the secretory vesicle or apical plasma membrane, and the membrane surrounding the secreted fat droplets is called the milk fat globule membrane (MFGM) [6]. MFGM is the main source of phospholipids in breast milk, consisting of glycerophospholipids and sphingomyelin (SM). The primary glycerophospholipids are phosphatidylethanolamine (PE), phosphatidylinositol (PI), phosphatidylserine (PS), and phosphatidylcholine (PC), while SM is the major sphingolipids [7].

PLs take around 0.2–1% of total milk lipid weight [8], and play significant roles in the physiological function and stability maintenance of MFGM. SM forms “lipid rafts” together with cholesterol to increase the rigidity of MFGM [9], while glycerophospholipids improve the fluidity of MFGM with a relatively higher degree of unsaturation [10], in a way that PLs are segregated between liquid-ordered domains and liquid-disordered phases. Early supplementation of PLs could improve cognitive development in animal models, manifesting as fewer errors and less respondence time in spatial T-maze tasks [11]. The intervention group also showed heavier brain weight, multiple brain areas with greater volumes, more gray and white matter, and increased PC-derived metabolites in the hippocampus [11]. Besides, milk PLs supplementation attenuated the severity of colitis by balancing goblet cell differentiation and reinforcing the mucus barrier in mice [12]. Moreover, milk phospholipids facilitated infants’ digestion as an essential dairy emulsifier [13] and also played a role in the establishment of gut microbiota [14].

The exclusive breastfeeding rate at 6 months has reached 74.9% in 2018 in China [15]. The role of human milk and breastfeeding in mother and child health is well recognized. Children who have access to breast milk for a longer duration have lower contagious morbidity and mortality [16] and better neurocognitive performance [17] than do those who are not breastfed or accept breast milk for shorter periods. These benefits of long-term breastfeeding may even persist into adulthood [3]. Considering the importance of human milk, research on human milk components, such as macronutrients [18], vitamins [19,20] and minerals [21], have been reported in China. However, studies on bioactive substances in breast milk are limited. Human milk is a dynamic system, where the quantification of PLs in breast milk has diverse results over lactation due to different detection techniques, gestation, the duration after postpartum, and other physiological and environmental factors. Meticulous data on human-milk composition, with appropriate ranges by geography and ethnicity, is needed for a better understanding of nutrition demands in lactating women and their infants, and strategies targeting on promoting optimal development in infants without access to breast milk. Therefore, the aim of this study was to determine the major PLs classes (PE, PI, PS, PC and SM) in breast milk during 0–400 days postpartum in six regions of China via high performance liquid chromatography coupled with an evaporative light scattering detector (HPLC-ELSD).

## 2. Materials and Methods

### 2.1. Participants and Human Milk Samples Collection

This study was part of the Maternal Nutrition and Infant Investigation (MUAI) registered at the China Clinical Trial Registry (ID# ChiCTR1800015387), which was designed to investigate the nutritional and bioactive components in cord blood, maternal blood and breast milk in healthy Chinese women. To enhance the representativeness of the sample, participants were recruited from Guangzhou, Chengdu, Changchun, Lanzhou, Shanghai and Tianjin which are, respectively, located in the southern, southwest, northeast, northwest, southeast and central parts of China. This study was approved by the Medical Ethics Research Committee of the School of Public Health, Sun Yat-sen University (Approval No. 017(2018)) and conducted in accordance with the Declaration of Helsinki. Written informed consent was obtained from all participants.

Lactating women aged between 21 to 44 with a full-term (37–42 weeks of pregnancy) and singleton delivery were recruited. Exclusion criteria were mothers with metabolic diseases, acute or chronic infectious diseases, serious heart/kidney diseases, or taking drugs that affect the metabolism of nutrients. In addition, mothers with infants who had Apgar score < 8, hereditary diseases, or lactation contraindications (such as phenylketonuria and galactosemia) were also excluded. Basic demographic information, height, pre-pregnancy and prenatal weight, medical history, pregnancy symptoms of mothers and newborns’ sex, weight, length, Apgar score and gestation were collected by trained investigators through questionnaires and the obstetric medical record system.

In this study, a total of 2270 breast milk samples were collected including 259 colostrum milk (0–5 days postpartum), 254 transitional milk (10–15 days), 630 early stage mature milk (40–45 days), 576 late stage mature milk (200–240 days) and 551 prolonged mature milk (300–400 days). The flowchart of this study was shown in Supplementary Figure S1. Under the instruction and guidance of our trained investigators, participants provided the sample at the second feeding in the morning during 9–11 a.m., where an electric pump was utilized to empty a single full breast. After completely blended, 15 mL aliquot was transferred to the lab under 4 °C within 6 h. All samples were stored under −80 °C before analysis.

### 2.2. Quantification of PLs

PLs standards including PE, PI, PS, PC and SM were all purchased from Sigma Company (purity for PE: 97%, PI: 99%, PS: 97%, PC: 99%, SM: 98%; St. Louis, MO, USA). Quantification was performed by HPLC-ELSD (Agilent 1260, Santa Clara, CA, USA) using an external standard method, which has been validated in our previous study [22]. The RSD% of five PLs classes examined sixteen times in eight days were all within 10.03%, which indicated good repeatability of this method. Six recovery experiments for each concentration level (50%, 100%, 150% of substrate concentration) were conducted over three days. The recoveries of five PLs classes and TPL ranged from 80.17% to 101.81% and 90.06% to 91.37%, respectively, which showed the good accuracy of this method. Therefore, the HPLC-ELSD method could meet the requirements for the quantification of PLs in human milk.

In brief, a 250 μL sample of breast milk in water (1:1, *v*/*v*) was vortexed in a 9.5 mL mixture of chloroform (HPLC grade, Sinopharm Chemical Reagent, Beijing, China) and methanol (HPLC grade, Thermo Fisher Scientific Company, Waltham, MA, USA; 2:1, *v*/*v*), followed by sonicating at 40 °C for 15 min. Then the sample was added to 2 mL of 5.43 mol/L NaCl and centrifuged at 2000 rpm for 5 min. The precipitation and supernatants were collected respectively. The supernatants were mixed with 7 mL of chloroform and methanol (6:1, *v*/*v*) and then added into precipitation for further extraction. The extraction was dried out by bath-type nitrogen blowing instrument at 40 °C and was dissolved in 250 μL of chloroform and methanol (9:1, *v*/*v*). The dissolved extraction was filtered by 0.2 μm PTFE membrane for HPLC-ELSD analysis.

Chromatographic separations were performed on a BETA SIL silica-100 column (200 × 3 mm, 3 μm, Thermo Fisher, Waltham, MA, USA). The column temperature was 40 °C and the injection volume was 25 μL. Solvent A was consisted of 87.5% chloroform/12.0% methanol/0.5% formic acid. Solvent B was consisted of 28% chloroform/60% methanol/12% formic acid. The pH of formic acid (HPLC grade, Anpel, Shanghai, China; 1 mol/L) was adjusted to 3 by triethylamine (HPLC grade, Sigma-Aldrich, St. Louis, MO, USA). Gradient conditions were as follows: time = 0 min, 100% solvent A and 0% solvent B; time = 20 min, 75% solvent A and 25% solvent B; time = 25 min, 60% solvent A and 40% solvent B; time = 25.1 min, 0% solvent A and 100% solvent B; time = 30 min, 0% solvent A and 100% solvent B; time = 31 min, 100% solvent A and 0% solvent B; time = 35 min, 100% solvent A and 0% solvent B; flow rate = 0.6 mL/min. The ELSD parameters was as follows: evap = 90 °C; neb = 40 °C, with a flow rate of N_2_ = 1.4 L/min.

### 2.3. Statistical Analysis

All data were analyzed using IBM SPSS Statistics 23.0 (SPSS, Inc., Chicago, IL, USA). Appropriate statistical analysis was performed after checking the normality of the distribution of the data and the equality of variances. We used Analysis of Covariance (ANCOVA) to compare the mean differences of PLs content during lactation in different multivariate models. *P*-values were based on two-sided tests and were considered significant at <0.05. The Bonferroni test was used for multiple comparisons with a corrected significant *p*-value corresponding to 0.005. The missing data of demographic characteristics and anthropometric information was deemed a system missing value. The total phospholipid (TPL) was the sum of the concentration of PE, PI, PS, PC and SM. Sub-group analysis was conducted in different regions where TPL concentration was compared over lactation by the Kruskal-Wallis test (Supplementary Figure S2). Correlations between maternal and infants’ factors and TPL concentration were also explored (Supplementary Table S1).

## 3. Results

### 3.1. Demographic and Anthropometric Characteristics

Mothers’ and infants’ anthropometric characteristics, as well as socio-demographic factors, were shown in Table 1. Participants’ pre-pregnancy BMI (21.04 ± 2.55 kg/m^2^), gestational weight gain (14.17 ± 3.96 kg) and infants’ birth weight (3.35 ± 0.49 kg), birth length (50.03 ± 2.16 cm) were all within normal range. The rate of vaginal delivery and female infants were 60.2% and 53.0%, respectively.

### 3.2. Total Phospholipid and Sub-Class Phospholipids in Human Milk

**Figure 1:**
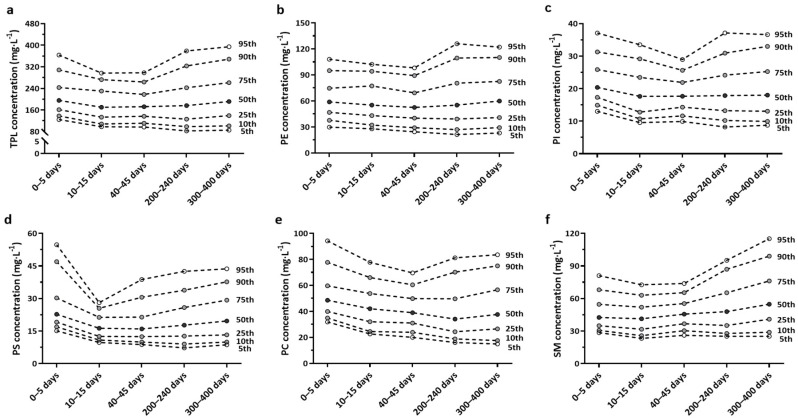
The percentile values of TPL concentration (**a**), PE concentration (**b**), PI concentration (**c**), PS concentration (**d**), PC concentration (**e**) and SM concentration (**f**) over lactation are shown. PC, phosphatidylcholine; PE, phosphatidylethanolamine; PI, phosphatidylinositol; PS, phosphatidylserine; SM, sphingomyelin; TPL, total phospholipid.

Figure 1 presented the percentiles of TPL and different classes of PLs over lactation periods, where graphical results described the distribution. The median (IQR) TPL content was in a range between 170.38 ± 96.52 mg/L to 195.69 ± 81.80 mg/L during lactation (25th percentile of TPL: 126.09–161.51 mg/L; 75th percentile of TPL: 217.70–261.42 mg/L) and was higher concentrated in colostrum milk and later stage of lactation (after 200 days postpartum) compared with that in the samples collected between 10 to 45 days post-partum. The distribution of five major sub-class PLs varied in different lactation stages (the medians ± IQR were: PE, 52.61 ± 29.05 to 59.95 ± 41.74 mg/L; PI, 17.65 ± 10.68 to 20.38 ± 8.55 mg/L; PS, 15.98 ± 9.02 to 22.77 ± 11.17 mg/L; PC, 34.13 ±25.33 to 48.64 ± 19.73 mg/L; SM, 41.35 ± 20.31 to 54.79 ± 35.26 mg/L). The 25th percentile (PE: 39.23–46.89 mg/L; PI: 12.81–17.35 mg/L; PS: 12.43–19.13 mg/L; PC: 24.36–39.96 mg/L; SM: 31.71–40.98 mg/L), 75th percentiles (PE: 69.29–82.59 mg/L; PI: 21.91–25.89 mg/L; PS: 21.34–30.30 mg/L; PC: 49.69–59.69 mg/L; SM: 52.02–76.24 mg/L) and other percentiles were also shown in detail.

### 3.3. Differences in Total Phospholipid and Sub-Class Phospholipids Concentration over Lactation in Chinese Human Milk

The differences in TPL and sub-class PLs concentration over lactation were examined in different multivariate models by ANCOVA (Table 2). After adjusting for socio-demographic factors and anthropometric characteristics of mothers and infants in model 1, the average TPL concentration was higher in colostrum (208.95 ± 5.03 mg/L) than that in mature milk of 40–45 days (182.47 ± 3.19 mg/L). Then higher TPL concentrations were observed in 200–240 days’ mature milk (195.02 ± 3.34 mg/L), and 300–400 days’ mature milk (210.50 ± 3.43 mg/L) compared with that in 40–45 days’ mature milk. Meanwhile, results varied in sub-class PLs. In detail, a much lower concentration of PE was observed in mature milk for 40–45 days (56.26 ± 1.13 mg/L), while PI, PS and PC were relatively higher during colostrum (PI: 21.91 ± 0.49 mg/L; PS: 26.61 ± 0.67 mg/L; PC: 52.28 ± 1.20 mg/L). However, SM aggregation was higher in later lactation periods (200–240 days: 53.33 ± 0.88 mg/L; 300–400 days: 60.58 ± 0.90 mg/L). Results were robust in model 2, in which we additionally adjusted participants’ areas and all other covariates in model 1 to reduce regional disparities.

**Figure 2:**
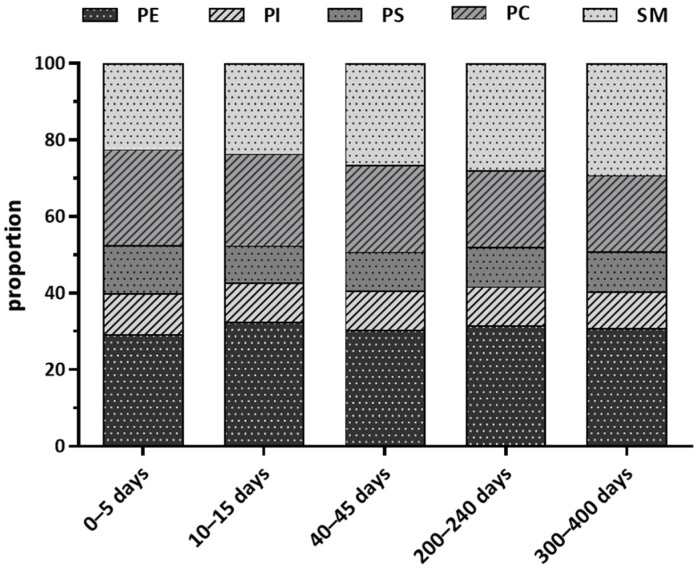
Proportion of components in total phospholipid at different lactation stage (%). PC, phosphatidylcholine; PE, phosphatidylethanolamine; PI, phosphatidylinositol; PS, phosphatidylserine; SM, sphingomyelin.

Furthermore, we described the proportion of components in TPL at different lactation stages in Figure 2. PE was the most abundant PLs constituent in human milk (ranging from 29.18% to 32.52%) across lactation. In comparison, PI and PS together accounted for around 20% (PI: ranged from 9.61% to 10.65%; PS: ranged from 9.65% to 12.73%). Moreover, PC constituted larger proportion in colostrum (25.04%) and transition milk (23.98%) than SM (colostrum: 22.39%; transition milk: 23.64%), but results were reversed in later lactation periods (PC vs. SM in mature milk of 40–45 days: 22.66% vs. 26.55%; 200–240 days: 19.97% vs. 27.97%; 300–400 days: 19.90% vs. 29.17%).

## 4. Discussion

In this study, we quantified five major sub-class PLs in a sample of 2270 human milk collected from six cities respectively located in the southern, southwest, northeast, northwest, southeast and central part of China. The optimized and validated HPLC-ELSD method is utilized for the determination of PLs. To our knowledge, this is the first cross-sectional study that explored the content of PLs over a plenty long lactation period (0–400 days postpartum) in a representative sample of the Chinese population, and our results update Chinese PLs data for human milk. TPL presented a “U” shape and sub-class PLs showed various fluctuations during lactating. PE, PC and SM were the dominant classes of PLs across lactation.

Human milk is a dynamic system and varied by stage of lactation. Consistent with the temporal change reported in a previous study [23], TPL concentration presented a “U” shape along lactation, higher concentrations were observed in colostrum and late lactational stages (200–400 days postpartum) compared with that in mature milk of 40–45 days. The decline during early lactation periods (around 0–90 days postpartum) was also observed in Wei et al.’s [24] study. However, some studies detected that TPL continued to decrease over the entire lactation period [25,26]. Higher aggregation of TPL content in early lactation is without dispute, and this may relate to maternal physical condition and the adaptive process during the physiological development of infants. Specifically, MFG size has been demonstrated to be negatively associated with TPL content [27], which indicates that a higher concentration of TPL in colostrum enables better dispersion of triacylglycerols in the form of smaller MFG with expanded surface area for anchoring of gastric lipase [28], thus facilitating fat digestion and absorption in newborns with poor pancreatic secretion accordingly [13]. Discrepancies in TPL concentration during the later lactation period may be due to different study populations and definitions of the lactation period. Studies mentioned above included both Asian [24,25] and Caucasian populations [26]. Furthermore, we defined three mature milk periods, namely the early stage mature milk (postpartum day 40 to 45), late stage mature milk (postpartum day 200 to 240) and the prolonged stage mature milk (postpartum day 300 to 400), which was consistent with the partitions used in Malaysian study [23]. However, the lactation stage was not specifically distinguished in the MING study [25] and the MISC cohort study [29]. Other possible explanations for the U-shape trajectory were the dramatic upregulation in the expression of genes associated with lipid metabolism and milk FA production in the initiation of human lactation [30], and the increased de novo synthesis in the mammary gland with the establishment of lactation [31,32].

China has vast geographical areas and multi-ethnic cultures, there are discrepancies in lifestyle behaviors and dietary patterns between regions. We examined our findings in different multivariate models and provided robust results of PLs fluctuation across lactation in Chinese human milk. We additionally gave detailed data on TPL concentration over lactation respectively in six cities in Supplementary Figure S2. TPL concentration in the early stages of lactation was higher than that in mature milk in Guangzhou, which was in accordance with our previous study [33] but was different from five other regions here. Some studies have reported the associations between maternal diet and human milk lipids and fatty acids composition [34,35,36]. For example, higher diet quality, reflected by the HEI-2010 score, was associated with lower total saturated fatty acids (SFAs) and higher poly-unsaturated fatty acids (PUFAs) in human milk [35]. Dietary protein was positively related to SFAs of PLs but negatively correlated with PUFAs of PLs [36]. Different food groups were also closely related to differential phospholipids [36]. As an important lipid component, milk PLs may also be affected by these factors. Although we tried to reduce regional disparities in our study, future studies should also aim to investigate cross-cultural risk factors of human milk components.

In general, sub-class PLs showed diverse changes along lactation. It is worthy to note that a similar pattern of five sub-class PLs was observed. PE was the most abundant sub-class PL identified in our study, although previous findings were contradictory [23,24,25,26]. PE has a higher degree of unsaturation than other sub-class PLs, acting as an important source of arachidonic acid (ARA, 20:4n-6) and docosahexaenoic acid (DHA, 22:6n-3) [37]. DHA and ARA are notably abundant in the central nervous system as structural components of membrane PLs and benefit the brain development of infants [38]. Although PC, rather than PE, and its metabolite lysophosphatidylcholine (LPC) are transferred to the brain via lipoprotein as a major carrier of ARA and DHA [39], infants could synthesize PC from PE in the liver [40]. Therefore, relatively higher PE and PC content in breast milk could ensure PUFA supplies for brain development in the early life period. Meanwhile, PC and PE are the first two dominant PLs in mammalian cells membrane [41], higher concentration of PE and PC in breast milk could provide enough membrane material to meet the rapid growth and development of infants’ organs after birth. Furthermore, PE and PC mainly esterify unsaturated fatty acids, to help of maintaining the stability of MFGM by increasing the fluidity of MFGM [42].

SM and PC were the second most rich PL constituents in human milk identified in our study, their temporary changes throughout lactation were exactly the opposite. SM and PC are choline-containing PLs and have been recognized as essential factors for optimum brain development of infants [43]. Although a large proportion of choline was provided by PC and SM through the placenta [44], water soluble choline was the major existing form in human milk [45], which could be explained by low or absent phosphatidylethanolamine N-methyl-transferase (PEMT) expression in placental tissue and fetal liver [46], but infants were able to endogenously produce PC after birth. Likewise, this could demonstrate why PC showed a decline in human milk. Moreover, SM could be synthesized from PC [41], also accounting for the diverse trend of SM and PC content in human milk. SM contributes to brain plasticity by being involved in myelination during the first two years postnatal [47]. SM is also an important component of the cell membrane [41], which forms lipid raft with cholesterol to help maintain the stability of the cell and engages in signal transduction [48].

PI and PS accounted for only a small portion of TPL, and studies about these two components’ impact on infants are limited. Current evidence showed that PS accounts for 13–15% of TPL in the human cerebral cortex [49] and participates in the activation of Akt, PKC, and Raf-1 signaling pathways, which is considered to stimulate neuronal survival, neurite growth and synaptogenesis [50]. PI is proven to be involved in signal transduction of the central nervous system and maintains the homeostasis of Ca^2+^ [51]. The physiological importance of PI and PS in children is worth further exploring.

Several limitations should be addressed in our study, which may be inherent to the observational design and general setting. Firstly, we only measured five major sub-class PLs without the identification of specific PL molecular structures. Although isomers may have an analogous physiological function accurate profiling of PL molecular species could provide a better understanding of human milk composition. Secondly, milk sample collection at one time point of one participant may limit the ability to speculate about long-term changes in the nutrient constituents during lactation periods. The multicentric recruitment allowed the inclusion of mothers from different parts of China and the presentation of representative PLs data of Chinese human milk. Besides, the normative milk collection method was delivered to participants by trained interviewers and consistent timing of collection was also required. Uniform and standardized analysis procedures minimized systematic and random errors in the assessment of PL profile in milk. Thirdly, diverse determinants may associate with the content and composition of breast milk, the present study did not evaluate all of them. However, we have adjusted participants’ social-demographical characteristics in multivariate models to analyze the variation. Moreover, only limited correlations between maternal and infants’ factors and human milk phospholipids concentration were observed (Supplementary Table S1), studies with longitudinal follow-up and rigorous bioinformatic tools to comprehensively explore determinants of milk composition, as well as strategies for protecting, promoting, and supporting breastfeeding are needed. Lastly, although our previous study profiled the phospholipidome in human milk samples [33], only subjects recruited in Guangzhou (a city in the southern part of China) were included. In this study, we described PLs concentration and composition of human milk in different lactational stages with a representative sample of the Chinese population by HPLC-ELSD. HPLC-ELSD has a similar response for molecules with similar structure and good compatibility with a much wider range of solvents and modifiers, making it a more suitable technique for the quantification of PLs mixture in large samples [52].

## 5. Conclusions

Phospholipids play a pivotal role in infant growth and neurodevelopment and have beneficial effects on aiding digestion. In the current study, we examined five major PL components, namely phosphatidylethanolamine, phosphatidylinositol, phosphatidylserine, phosphatidylcholine and sphingomyelin in Chinese human milk from postpartum day 0 to day 400. Human milk TPL presented a “U” shape and sub-class PLs showed various fluctuations during lactating. PE, PC and SM were the dominant classes of PLs and took a proportion of 29% to 33%, 20% to 25%, and 22% to 29%, respectively, across lactation. Our study updates Chinese PLs data in human milk, which provides evidence for better defining nutrient reference values for infants, and further contributes to developing integrated strategies to support the development of secure and effective human-milk surrogates for children who do not have access to breast milk.

## Figures and Tables

**Table 1:** Demographic and anthropometric characteristics of lactating women and corresponding infants.

Characteristics	0–5 Days (*n* = 259)	10–15 Days (*n* = 254)	40–45 Days (*n* = 630)	200–240 Days (*n* = 576)	300–400 Days (*n* = 551)
Mothers					
Age (years)	29.18 ± 3.47	29.06 ± 3.27	29.60 ± 3.29	29.96 ± 3.43	30.26 ± 3.43
Pre-pregnancy BMI (kg/m^2^)	21.66 ± 3.13	21.50 ± 2.99	21.42 ± 2.97	20.80 ± 2.56	21.01 ± 2.77
Gestationalweight gain (kg)	14.60 ± 4.58	14.85 ± 4.68	14.72 ± 4.92	14.52 ± 5.41	14.21 ± 5.35
Delivery mode					
Vaginaldelivery	155 (61.5%)	151 (60.6%)	352 (56.3%)	361 (63.1%)	332 (60.8%)
Caesarean	97 (38.5%)	98 (39.4%)	273 (43.7%)	211 (36.9%)	214 (39.2%)
Infants					
Birth weight (kg)	3.38 ± 0.41	3.39 ± 0.41	3.35 ± 0.40	3.52 ± 0.67	3.65 ± 0.82
Birth length (cm)	49.84 ± 1.49	49.97 ± 1.36	49.84 ± 1.49	53.41 ± 7.76	54.42 ± 9.59
Infant gender					
Female	125 (49.6%)	125 (50.2%)	312 (49.9%)	282 (49.3%)	264 (49.4%)
Male	127 (50.4%)	124 (49.8%)	313 (50.1%)	290 (50.7%)	282 (50.6%)

Data are expressed as mean ± SD for continuous variables and *n* (%) for categorical variables.

**Table 2:** Differences of TPL and sub-class PLs concentration in human milk over lactation (mg/L).

PLs	0–5 Days (*n* = 259)	10–15 Days (*n* = 254)	40–45 Days (*n* = 630)	200–240 Days (*n* = 576)	300–400 Days (*n* = 551)
Model 1					
PE	61.85 ± 1.78 ^ab^	61.46 ± 1.79 ^ab^	56.26 ± 1.13 ^b^	62.67 ± 1.18 ^a^	65.25 ± 1.22 ^a^
PI	21.91 ± 0.49 ^a^	19.12 ± 0.49 ^b^	18.48 ± 0.31 ^b^	19.34 ± 0.32 ^b^	19.75 ± 0.33 ^b^
PS	26.61 ± 0.67 ^a^	17.49 ± 0.67 ^d^	18.88 ± 0.42 ^cd^	20.28 ± 0.44 ^c^	22.09 ± 0.45 ^b^
PC	52.28 ± 1.20 ^a^	44.82 ± 1.21 ^b^	41.45 ± 0.76 ^bc^	39.40 ± 0.80 ^c^	42.83 ± 0.82 ^b^
SM	46.30 ± 1.33 ^c^	43.26 ± 1.33 ^c^	47.41 ± 0.84 ^c^	53.33 ± 0.88 ^b^	60.58 ± 0.90 ^a^
TPL	208.95 ± 5.03 ^ab^	186.15 ± 5.06 ^bc^	182.47 ± 3.19 ^c^	195.02 ± 3.34 ^b^	210.50 ± 3.43 ^a^
Model 2					
PE	61.23 ± 1.77 ^ab^	60.85 ± 1.78 ^ab^	56.39 ± 1.12 ^b^	62.78 ± 1.17 ^a^	65.53 ± 1.20 ^a^
PI	21.74 ± 0.48 ^a^	18.95 ± 0.48 ^bc^	18.52 ± 0.31 ^c^	19.37 ± 0.32 ^bc^	19.83 ± 0.33 ^b^
PS	26.55 ± 0.67 ^a^	17.44 ± 0.67 ^d^	18.89 ± 0.42 ^cd^	20.86 ± 0.44 ^c^	22.12 ± 0.45 ^b^
PC	52.14 ± 1.20 ^a^	44.68 ± 1.21 ^b^	41.48 ± 0.76 ^bc^	39.43 ± 0.80 ^c^	42.90 ± 0.82 ^b^
SM	46.54 ± 1.32 ^c^	43.49 ± 1.33 ^c^	47.36 ± 0.84 ^c^	53.28 ± 0.88 ^b^	60.47 ± 0.90 ^a^
TPL	208.20 ± 5.03 ^ab^	185.41 ± 5.05 ^c^	182.64 ± 3.19 ^c^	195.15 ± 3.33 ^bc^	210.85 ± 3.42 ^a^

Values were least square means in ANCOVA, different superscript letters (a–d) indicated significant differences (*p* < 0.005 with Bonferroni correction); Model 1: adjusted for mother’s age (continuous), pre-pregnancy BMI (continuous), gestational weight gain (continuous), delivery mode (natural delivery or cesarean), infant gender (female or male), birth weight (continuous) and birth length (continuous); Model 2: adjusted for all variables in Model 1, and participants areas (Chengdu, Guangzhou, Changchun, Lanzhou, Shanghai or Tianjin) additionally; Abbreviations: PC, phosphatidylcholine; PE, phosphatidylethanolamine; PI, phosphatidylinositol; PLs, phospholipids; PS, phosphatidylserine; SM, sphingomyelin; TPL, total phospholipid.

## Data Availability

Data described in the manuscript, code book, and analytic code will not be made available because approval has not been granted by study participants.

## Supplementary Materials

The following supporting information can be downloaded at: https://www.mdpi.com/article/10.3390/nu14081539/s1, Table S1: The correlations between human milk phospholipids concentration and baseline characteristics; Figure S1: Flowchart of the study population; Figure S2: Differences in TPL concentration over lactation in Chengdu (A), Guangzhou (B), Changchun (C), Lanzhou (D), Shanghai (E) and Tianjin (F).

## Abbreviations

MFG, milk fat globule; PLs, phospholipids; HPLC-ELSD, high performance liquid chromatography coupled with evaporative light scattering detector; MFGM, milk fat globule membrane; SM, sphingomyelin; PE, phosphatidylethanolamine; PI, phosphatidylinositol; PS, phosphatidylserine; PC, phosphatidylcholine; TPL, total phospholipid; PUFAs, polyunsaturated fatty acids; SFAs, saturated fatty acids; ARA, arachidonic acid; DHA, docosahexaenoic acid; LPC, lysophosphatidylcholine; PEMT, phosphatidylethanolamine N-methyl-transferase.
