# Supplementary material for: Lactational Changes of Phospholipids Content and Composition in Chinese Breast Milk
Source: Nutrients. 2022 Apr 7;14(8):1539. doi: 10.3390/nu14081539 (PMC9030290; doi:10.3390/nu14081539)
Supplement: Supplementary file 1 [file nutrients-14-01539-s001.zip › nutrients-1631941-supplementary/Supplementary Figure S2.pptx]

## Slide 1
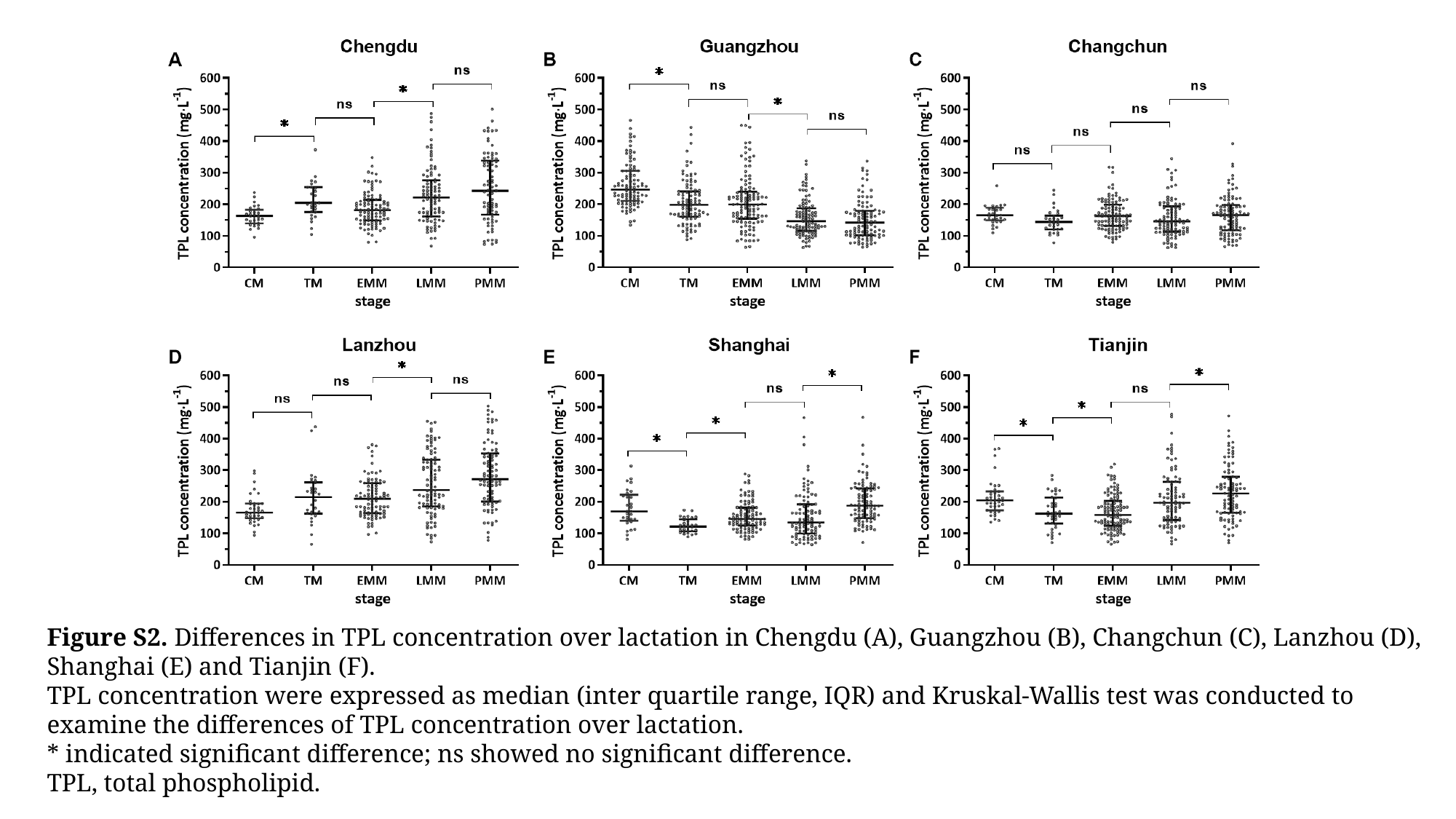

Figure S2. Differences in TPL concentration over lactation in Chengdu (A), Guangzhou (B), Changchun (C), Lanzhou (D), Shanghai (E) and Tianjin (F).
TPL concentration were expressed as median (inter quartile range, IQR) and Kruskal-Wallis test was conducted to examine the differences of TPL concentration over lactation.
* indicated significant difference; ns showed no significant difference.
TPL, total phospholipid.
