# Supplementary material for: Lactational Changes of Phospholipids Content and Composition in Chinese Breast Milk
Source: Nutrients. 2022 Apr 7;14(8):1539. doi: 10.3390/nu14081539 (PMC9030290; doi:10.3390/nu14081539)
Supplement: Supplementary file 1 [file nutrients-14-01539-s001.zip › nutrients-1631941-supplementary/Supplementary Table S1.pdf]

Table S1. The correlations between human milk phospholipids concentration and baseline characteristics

	PE	PI	PS	PC	SM	TPL
Mothers						
Age (years)	0.008	0.014	-0.014	-0.006	0.028	0.008
Pre-pregnancy BMI (kg/m^2)	0.013	0.014	0.019	0.031	0.040	0.027
Gestational weight gain (kg)	-0.040	-0.037	-0.054*	-0.037	-0.055*	-0.049*
Delivery mode	-0.017	0.009	-0.052*	-0.017	-0.037*	-0.022
Infants						
Birth weight (kg)	-0.029	-0.064*	-0.036	-0.068*	0.002	-0.037
Birth length (cm)	-0.061*	-0.109*	-0.047*	-0.119*	0.003	-0.066*
Infant gender	-0.010	-0.001	0.002	-0.013	0.001	-0.006

Correlations between concentration of human milk phospholipids and baseline characteristics were analyzed via Spearman correlations (categorical variables) and Pearson correlation (continuous variables).

*indicated p <0.05.

Abbreviations: PC, phosphatidylcholine; PE, phosphatidylethanolamine; PI, phosphatidylinositol; PS, phosphatidylserine; SM, sphingomyelin; TPL, total phospholipid.
