# Supplementary figures and images for: Lactational Changes of Phospholipids Content and Composition in Chinese Breast Milk
Source: Nutrients. 2022 Apr 7;14(8):1539. doi: 10.3390/nu14081539 (PMC9030290; doi:10.3390/nu14081539)

## Slide 1
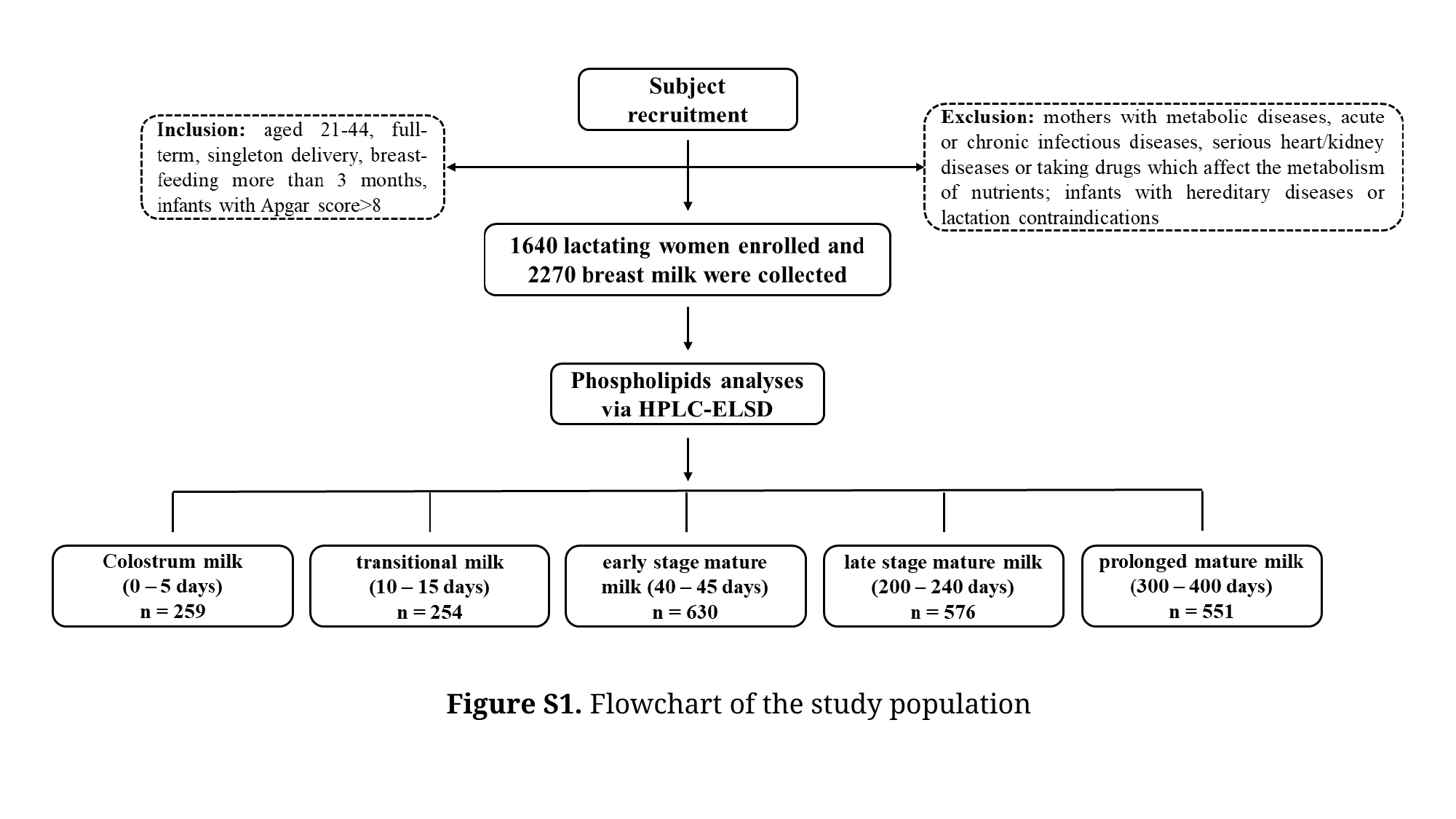

Figure S1. Flowchart of the study population

Supplement: Supplementary file 1 [file nutrients-14-01539-s001.zip › nutrients-1631941-supplementary/Supplementary Figure S1.pptx]
